# Orthobiologic Injections for the Treatment of Hip Osteoarthritis: A Systematic Review

**DOI:** 10.3390/jcm11226663

**Published:** 2022-11-10

**Authors:** Marco Zaffagnini, Angelo Boffa, Luca Andriolo, Federico Raggi, Stefano Zaffagnini, Giuseppe Filardo

**Affiliations:** 1 Clinica Ortopedica e Traumatologica 2, IRCCS Istituto Ortopedico Rizzoli, 40136 Bologna, Italy; 2 Applied and Translational Research (ATR) Center, IRCCS Istituto Ortopedico Rizzoli, 40136 Bologna, Italy

**Keywords:** osteoarthritis, hip, orthobiologics, platelet rich plasma (PRP), bone marrow aspirate concentrate (BMAC), micro-fragmented adipose tissue (MF-AT), amniotic suspension allograft (ASA), mesenchymal stromal cells (MSCs), injective

## Abstract

The use of orthobiologics is gaining increasing interest as a minimally invasive treatment for hip osteoarthritis (OA). The aim of this study was to investigate the evidence about the safety and efficacy of these products. A systematic review of the literature was performed according to the PRISMA and Cochrane guidelines. The study quality was assessed using the RoB 2.0 for randomized controlled trials (RCTs) and the modified Coleman Methodology Score (mCMS) for all studies. A total of 20 clinical studies (735 patients) was identified, 12 on PRP injections and eight on cell-based therapies (five from bone marrow, two from adipose tissue, and one from amniotic fluid). The publication trend increased over time, with over 50% of articles published from 2019. The literature analysis showed only six RCTs, all on PRP injections. The mCMS showed an overall fair methodology (mean score 59.4). While the number of studies and their methodology are still limited, the available evidence suggests safety and overall promising results, with the treatment success being inversely proportional to the severity of OA. Further high-level controlled trials are needed before drawing more definitive conclusions on the real potential of orthobiologics for the injective treatment of patients affected by hip OA.

## 1. Introduction

Osteoarthritis (OA) is one of the most frequent degenerative joint diseases, characterized by progressive deterioration and loss of articular cartilage with concomitant structural and functional changes in the affected joint [1]. The hip is one of the most commonly involved joints and it has been estimated that 9.2% of people >45 years old have symptomatic hip OA [2], characterized by pain, stiffness, and loss of mobility often associated with functional limitation [3,4]. Currently, several conservative strategies can be used for the management of hip OA including weight loss, activity modification, physical treatments, oral medications, and intra-articular injections with corticosteroids or hyaluronic acid (HA) [5,6,7,8]. However, these treatments mainly provide symptom relief rather than disease-modifying changes, so often total hip arthroplasty (THA) is required as the definitive treatment for hip OA. THA is a particularly successful surgery although it represents a major intervention and is associated with rare but important risks of complications and unsatisfactory results, especially in the youngest patients [9,10]. Thus, in order to avoid or delay the need for THA, it is important that new non-operative options are explored for the management of patients with hip OA [11].

The use of orthobiologics is gaining increasing interest as a mini-invasive treatment option for OA aiming at reducing symptoms, improving function, possibly preventing OA progression, and delaying the need for total hip replacement. Numerous orthobiologic products are currently applied in clinical practice as injective treatments, ranging from blood derivatives to cellular therapies [12]. Among these products, platelet-rich plasma (PRP) has gained particular attention due to the high concentration of growth factors, cytokines, and bioactive molecules stored in platelet α-granules, being involved in both healing processes, immunoregulation, and inflammation modulation [13,14]. More recently, cell-based therapies have been introduced in clinical practice to exploit the potential of mesenchymal stromal cells (MSCs) [15,16]. These cells are multipotent progenitor cells able to differentiate into several different lineages including osteogenic, chondrogenic, adipogenic, and myogenic cell lines. Moreover, MSCs showed immunomodulatory and anti-inflammatory actions, through direct cell-to-cell interaction or secretion of bioactive factors. However, regarding the clinical results of these treatments, most of the literature attention relies on findings deriving from knee OA research, while the experiences reported on other joints are more scattered. A state-of-the-art literature analysis is needed to understand the current evidence on the clinical benefits of these treatments for hip OA [16,17], a growing application of orthobiologics in clinical practice.

The aim of this systematic review was to investigate the evidence about the safety and efficacy of the use of orthobiologic injections for the treatment of hip OA.

## 2. Materials and Methods

### 2.1. Search Strategy and Article Selection

A systematic review of the literature was performed on the use of orthobiologics as injective treatment for hip OA. This study was registered on the international prospective register of systematic reviews (PROSPERO registration number: 2022 CRD42022315512) [18,19].

A literature search was conducted on 12 September, 2022 on three electronic databases (PubMed, Embase, and Web of Science), with no time limitation and without any filters, using the following string: “(Orthobiologic* OR biologic* OR platelet rich plasma OR PRP OR plasma rich in growth factors OR PRGF OR platelet derived growth factor OR platelet derived OR platelet gel OR platelet concentrate OR PRF OR platelet rich fibrin OR ACP OR autologous conditioned plasma OR APS OR autologous protein solution OR platelet lysate OR prolotherapy OR MSCs OR stem cells OR stromal cells OR progenitor cell OR bone marrow concentrate OR bone marrow aspirate concentrate OR BMAC OR micro-fra* adipose tissue OR microfra* adipose tissue OR stromal vascular fraction OR SVF OR amniotic suspension allograft OR ASA OR placenta* OR umbilical cord OR amnio*) AND (hip) AND (osteoarthritis or OA or cartilage degeneration or cartilage lesion)”.

**Figure 1:**
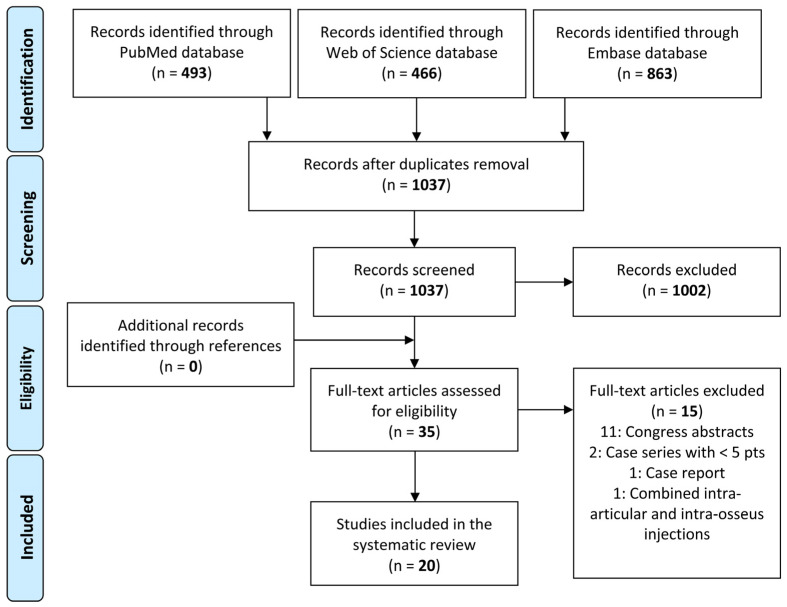
PRISMA (Preferred Reporting Items for Systematic Reviews and Meta-Analyses) flowchart of the study selection process.

According to the Preferred Reporting Items for Systematic Reviews and Meta-Analysis (PRISMA) and Cochrane guidelines [20], the article selection (Figure 1) and data extraction process were conducted separately by two authors (MZ and AB). The initial title and abstract screenings were made using the following inclusion criteria: clinical studies (at least five patients) of any level of evidence, written in the English language, and evaluating the intra-articular use of orthobiologics for the injective treatment of hip OA. Exclusion criteria consisted of articles written in other languages, literature reviews, preclinical (animal) studies, basic science in vitro articles, case series (less than five patients), case reports, congress abstracts, and studies on joint diseases different from OA. In the second step, full texts of the selected articles were screened, with further exclusion according to the previously described criteria. Additionally, all references from the selected papers and previously published relevant reviews were also screened. Two investigators reviewed each article (MZ and AB), and any discrepancies between them were resolved by discussion and consensus with a third author (LA).

### 2.2. Data Extraction, Outcome Measurement, and Quality Assessment

For the included studies, relevant data were extracted from article texts, tables, and figures, and then summarized and analyzed according to the purpose of the present work. In particular, the following data were collected: year of publication, study design, treatment type and schedule, details of the orthobiologic product used, number of evaluated patients, patient characteristics, hip OA grade, follow-up length, evaluation methods, main results, failures, adverse events, and funding sources when available. The efficacy of orthobiologic injective therapy in hip OA was evaluated by summarizing the reported benefits and evaluating the scores used, while the safety of the procedures was evaluated by identifying the reported side effects. 

The quality of the included studies was assessed by two separate authors (MZ and AB) using the Cochrane Collaboration Risk of Bias 2.0 tool (RoB 2.0) for RCTs and the modified Coleman Methodology Score (mCMS) for all studies [21]. The mCMS score ranges from 0 to 100, with a higher score reflecting higher quality. The final score was categorized as excellent (85–100 points), good (70–84 points), fair (55–69 points), and poor (<55 points). In case of disagreement between the two authors, divergences were discussed with a third author (LA), and a consensus was reached.

## 3. Results

### 3.1. Article Selection and Characteristics

After duplicates were removed, the initial search identified 1037 records, whose abstracts were screened and selected according to the inclusion/exclusion criteria for a total of 35 articles assessed for eligibility. No articles were identified through the reference lists. Fifteen studies were excluded after full-text evaluation: 11 congress abstracts, two case series that considered less than five patients, one case report, and one study that combined intra-articular and intra-osseous injections. Thus, a total of 20 clinical studies focusing on orthobiologic approaches for the management of hip OA were included in this systematic review [22,23,24,25,26,27,28,29,30,31,32,33,34,35,36,37,38,39,40,41].

Among the included studies, the evaluation by study type showed nine prospective case series, six RCTs, two retrospective case series, two prospective comparative studies, and one retrospective comparative study. Different orthobiologic products were investigated: 12 studies evaluated PRP and eight evaluated cell-based therapies, obtained from iliac crest bone marrow in five studies, from abdominal adipose tissue in two, and from amniotic fluid in one. A total of 735 patients affected by hip OA and treated with orthobiologic injections were evaluated: 502 were treated with PRP injections, 153 with adipose tissue-derived products, 80 with bone marrow-derived products, and 10 with amniotic suspension allograft (ASA).

**Figure 2:**
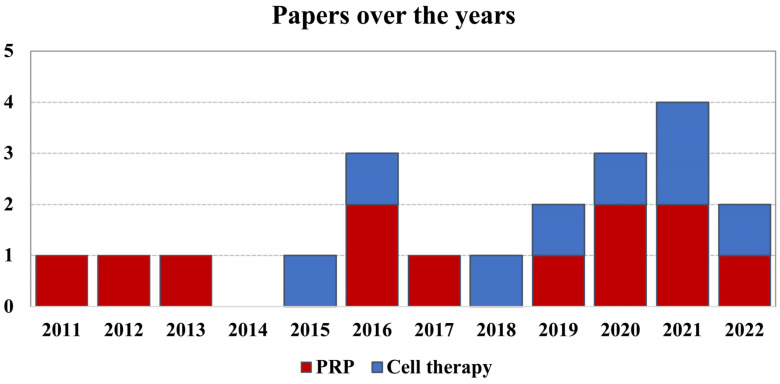
Number of articles published over time on the treatment of hip OA with orthobiologics.

Since the first report in 2011, the publication trend increased over time, with over 50% of articles published from 2019 (Figure 2).

Among the included studies, nine studies specified the failure of previous conservative treatments as inclusion criteria, while the other eleven studies did not report this aspect. The trial duration varied from 4 to 40 months of follow-up, with an average of 13.9 months. The injections were performed under guidance in 18 studies (ultrasonography in 15, fluoroscopy in two, both guidance methods in one), while guidance was not used in two studies. The visual analog scale (VAS) for pain (16 articles), the Harris Hip Score (HHS, 14 articles), and the Western Ontario and McMaster University Osteoarthritis Index (WOMAC, 12 articles) were the most commonly used scores. Other scores, such as the Numeric Pain Rating Scale (NRPS), the Pain Disability Quality-Of-Life Questionnaire (PDQQ), the Hip Outcome Score-Activities of Daily Living (HOS-ADL), the International Hip Outcome Tool (iHOT), the Short Form Health Survey 12 (SF-12), and the Single Assessment Numeric Evaluation (SANE) were also used in some studies. 

**Figure 3:**
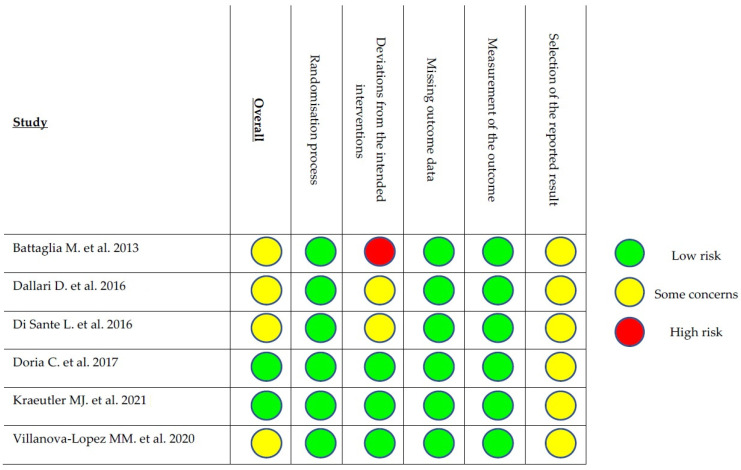
Assessment of the risk of bias for RCTs [26,27,28,29,30,31].

The evaluation with the mCMS showed an overall fair methodology of the included studies, with an average score of 59.4 points out of 100 (range 41–80). The risk of bias of the RCTs presented some concerns in four studies and was low in two studies. Details are reported in Figure 3. 

Among the included articles, 10 studies declared no funding, and six received a financing source. The remaining four articles did not report such data. 

Table 1 shows the number of patients from each study classified by Kellgren–Lawrence OA grade and Tönnis OA grade, respectively.

### 3.2. Orthobiologic Products

Regarding PRP characteristics, all 12 studies evaluated the use of autologous PRP, with one study also reporting the use of cordonal PRP (C-PRP) [33]. The concentration of platelets of the injected PRP was reported in seven studies, ranging from 1.7 to 6.0 higher than the whole blood concentration. Ten studies described the type of PRP based on leukocytes concentration: five studies used a leukocyte-poor PRP (LP-PRP), four studies used a leukocyte-rich PRP (LR-PRP) with a leukocyte concentration ranging from 3.9 to 8.3 × 10^3^/μL, and one study compared an autologous PRP rich in leukocytes versus a C-PRP without leukocytes. Only one study performed a quantification of cytokines in the injected PRP, evaluating the levels of proinflammatory and anti-inflammatory markers [27]. PRP was activated by calcium chloride in six articles, in one by calcium gluconate; in one it was not activated, while five articles did not describe the activation. PRP was cryopreserved in four studies, used fresh in three studies, while this aspect was not described in the other five studies. The injection amount was reported in every study with a range from 3 mL to 8 mL (median 5 mL). The most common injection schedule was three injections (nine articles), followed by one injection (two articles) and two injections (one article). In the studies with multiple injections, PRP was administered with one-week interval in six articles, two-week interval in three articles, while in one article the interval ranged from one to two weeks. Further details on the characteristics of the different PRPs used are reported in Table 2.

Regarding the eight studies on cell-based therapies, the injected product was autologous in seven studies and homologous in one study. Cell-based products produced at the point of care were used in six studies, while expanded MSCs were used in two studies. In particular, bone marrow aspirate concentrate (BMAC) injections were evaluated in three studies, bone marrow MSCs (BMSCs) injections in two studies, micro-fragmented adipose tissue (MF-AT) injections in two studies, and ASA injection in one study. The number of nucleated cells in the injected product was reported only in the two studies on BMSCs: in one study 20 × 10^6^ expanded BMSCs and in one study 5 × 10^5^ BMSCs/Kg/bw were injected [34,35]. The injection amount was reported in seven studies with a range from 4 mL to 12 mL. The most common injection protocol was a single injection administration (seven studies), while three injections of BMSCs were used in one study, with an interval of one week between the injections. The expression of surface markers and microbial contamination were investigated in the two studies on BMSC injections [34,35], while the hematology analysis to obtain a complete blood cell count of BMAC was investigated in only one study [38]. Further details on the characteristics of the different cell-based therapies used are reported in Table 3.

### 3.3. Safety

The safety of orthobiologic injections for the treatment of hip OA was documented by 19/20 studies. No severe adverse events occurring during harvest procedures, injective treatment, and post-injective follow-up periods were reported for both PRP and cell therapy approaches. Regarding mild adverse events, episodes of transient pain and joint discomfort at the injection site soon after the injection, which then spontaneously resolved, were reported. One study on PRP reported a superficial hematoma during hip injection due to transitional damage of a peripheral branch of the great saphenous vein, which spontaneously resolved in two weeks [26]. One study documented a mild rush after PRP injection, which disappeared spontaneously, and for the authors, it was not clearly related to the treatment [23]. Finally, one study on MF-AT reported an organized hematoma on the abdomen after the adipose tissue harvesting [37].

### 3.4. Clinical Efficacy

The main finding of the included studies was an overall improvement in pain and function in hip OA patients treated with orthobiologic injections, with similar results obtained for the different types of products. Improvements were observed mostly during the first 6 months after the injections, remaining stable throughout the follow-up periods, although some authors reported a gradual worsening of clinical outcomes toward the end of the follow-up [26,27,33]. Six RCTs investigated the comparison between PRP and HA injections, reporting controversial results. Among these, three studies were not able to prove an overall superiority of PRP over HA [26,29,30], two studies reported better results in the PRP group in terms of clinical improvement and delay of the need for THA [27,31], while one study documented a better clinical outcome for patients treated with HA injections [28]. One study analyzed two different PRPs in a comparative match-paired analysis, demonstrating no significant differences between autologous PRP and cordonal PRP injections, although the results were influenced by OA severity, with cordonal PRP showing more benefits when advanced OA cases were excluded [33]. The role of OA severity was also underlined in six studies (five on PRP and one on BMAC), all showing better clinical results after injections in patients with early or moderate OA compared with those with severe OA [23,24,26,27,30,38]. One study analyzed the number of patients who responded to the treatment in relation to the platelet concentration of the injected PRP, reporting that responders had higher platelet concentrations compared with non-responders [30]. Finally, the possibility to improve the results by combining different products was investigated in three studies [27,32,41]. Two studies did not prove a clear superiority of the combination of PRP and HA with respect to PRP or HA alone [27,32]. Similarly, one study did not demonstrate clear overall differences between the combination of MF-AT and PRP compared to MF-AT alone in the treatment of hip OA patients [41].

Failure of the treatments was documented in 16 studies, for a total of 839 patients evaluated. Patients that received a surgical treatment were 61 (7.2%); of these, 57 were treated with THA, one of these was treated for a fracture in an accident, two were treated with partial hip resurfacing, one with a mini-open surgery not specified, and one with arthroscopic treatment.

## 4. Discussion

The main finding of this systematic review is that there is increasing attention on orthobiologic injections for the treatment of hip OA. While the number of studies and their methodology is still limited, the available evidence suggests safety and overall promising results of orthobiologic injective treatments for hips affected by OA.

PRP and cell-based approaches gained significant interest due to the development of new promising products to address OA, especially thanks to the numerous studies derived from the knee OA research [42]. In particular, PRP has been widely investigated for knee OA, with several RCTs and meta-analyses demonstrating the superiority over placebo and other common injectable options such as corticosteroids or viscosupplementation [17]. PRP research for hip OA is less conspicuous, with 12 available clinical studies of which only six RCTs. A recent meta-analysis tried to summarize the clinical results of PRP injections for hip OA treatment, suggesting that PRP was effective in reducing pain and improving the function of the hip joint [43]. On the other side, another recent meta-analysis was not able to demonstrate the superiority of PRP injections over HA in hip OA, reporting similar results between the two treatments in short-term clinical outcomes [16]. However, the current literature is characterized by a high heterogeneity of the used products, which reduces the strength and evidence of the available meta-analyses. In fact, this systematic review underlined the remarkable differences among the included studies in the used PRPs in terms of preparation methods, composition, platelet and leukocyte concentration, activation method, and injection schedules. In particular, autologous PRPs were used in 12 studies, with one of these studies also investigating the use of homologous PRP (cordonal). Looking at studies reporting further details on PRP use, PRPs were with leukocytes in six studies and without leukocytes in five studies, the platelet concentrations ranged from 1.7 to 6 times higher compared to the whole blood, PRP was cryopreserved in four studies and fresh in three studies, and the activation of the PRP with exogenous activators was described in seven studies. Pooling different PRP products, each documented by sparse data, is not ideal from a methodological point of view and could offer weak results [44]. Therefore, the results of the available meta-analyses on PRP use for hip OA should be interpreted carefully in light of the heterogeneity and limitations of the field. Thus, while a statistical analysis bears the risk of misleading conclusions, the systematic review of all orthobiologic injective approaches allowed to offer a clear picture of this evolving field.

Among the different aspects of PRP injections, the concentration of leukocytes is one of the most debated. Some preclinical evidence suggests that leukocytes may be deleterious and impair the overall effect of PRP, while other findings support their use due to the release of beneficial cytokines [45,46]. Clinical evidence on the influence of leukocytes on PRP efficacy for OA joints is still limited, with only one RCT recently published for knee OA patients reporting comparable results between LR-PRP and LP-PRP in terms of clinical outcomes, adverse events, and failures [47]. For the hip joint, no direct comparisons have been made between the two PRP types, although the indirect comparison in the previously cited meta-analysis suggested a higher pain reduction in patients treated with LP-PRP [43]. Considering the high heterogeneity of the included studies and the limitations ascribable to a sub-group analysis, future high-level studies should investigate the role of leukocytes and other aspects in order to optimize the use of PRP for the treatment of hip OA.

This systematic review also showed an increasing interest in cell-based products for the treatment of hip OA, with eight clinical studies published since 2015. The growing interest in these products is due to the biological potential of MSCs, able to differentiate in several cells’ lines as well as to their paracrine action [48,49]. Immunomodulatory and anti-inflammatory action may positively affect joint homeostasis and eventually reduce pain and improve joint function [50,51]. Thanks to these properties, cell-based products also demonstrated to induce disease-modifying effects in OA animal models, slowing down the progression of cartilage degeneration [52]. This favored the application of cell-based therapies to address cartilage pathologies, including the intra-articular treatment of hip OA.

The most investigated cell-based products for hip OA were bone marrow-derived products, with three studies on the point of care BMAC and two studies on cultured-expanded BMSCs. Two studies analyzed adipose tissue-derived products, while only one study was on an amniotic-derived product. Although these studies described the safety and efficacy of cell therapies for hip OA treatment, their level of evidence is very low, being all prospective or retrospective case series without a comparison arm, except for the study of Heidari et al. [41], which compared the combined use of MF-AT and PRP versus the MF-AT approach alone in a non-randomized prospective study. Moreover, as for PRP, clinical studies on cell therapies are characterized by a high heterogeneity relative to the different MSC sources, MSC doses, injection schedules, and manufacturing processes, impairing the possibility to perform a meta-analysis. Therefore, the available evidence on cell therapies for hip OA is still limited, and the use of these products in clinical practice is still not supported by the literature with robust studies clearly confirming their benefits and superiority versus other treatments. 

Future high-level studies should better investigate the real potential of cell therapies and PRP, also comparing these products with the placebo effect. In fact, intra-articular injections are characterized by a significant placebo effect, which plays an important role, especially for new attractive products such as orthobiologics. A recent systematic review and meta-analysis of RCTs on knee OA injections demonstrated a significant placebo effect in terms of pain reduction and function improvement for up to 6 months [17,53]. Therefore, future randomized placebo-controlled studies should investigate the real therapeutic potential of orthobiologics for the injective treatment of hip OA. Further studies also should investigate possible factors that could influence the efficacy of orthobiologic injections for hip OA. Among these, the severity of hip OA has been already analyzed by six of the included studies in this systematic review, showing that the success of the treatment is inversely proportional to the severity of OA. In fact, patients with early or moderate OA reported better clinical results after the injections compared to those with severe OA [23,24,26,27,30,38]. These results confirmed what has already been demonstrated for orthobiologic injections in knee OA patients, with a lower efficacy obtained in knees with Kellgren–Lawrence 3 or 4 [45,54]. This and other aspects of the treated patients and of the injected products should be investigated to optimize the use of orthobiologics for hip OA.

The limitations of this systematic review reflect the limitations of this field. The literature analysis showed that clinical studies on orthobiologic injections to address hip OA are few and characterized by a low-level of evidence and high heterogeneity. Only six RCTs were conducted, without a placebo-controlled trial. These studies were all on PRP injections, while evidence from RCTs on cell-based therapies is lacking. Moreover, there are not enough stratified and homogeneous data based on the type of injected product, making it difficult to merge and compare clinical results, thus impairing the possibility to perform a reliable meta-analysis to draw clear conclusions. Accordingly, it was not appropriate to proceed with the data analysis, and the systematic review rather offered a state-of-the-art picture of the field. Aligned with this aim, the methodology of the selected studies was evaluated with the RoB 2.0 and mCMS, which confirmed the limited quality of the literature despite the RCTs available. Similarly, the included studies did not always report the exact number of adverse events and also used different definitions, hindering the possibility to obtain an accurate adverse event rate. Furthermore, some studies reported sponsor funding, which could affect the final results, underlying the need for independent research efforts to confirm these findings. Finally, the relatively short follow-up in most of the included studies leaves concerns regarding the durability of the treatment results with PRP and cell-based therapies for hip OA. Albeit being considered minimally invasive, these treatments still require a blood harvest or a surgical approach, and results should prove significant and long-lasting to justify their use versus less invasive conservative treatments.

## 5. Conclusions

There is increasing attention on orthobiologic injections for the treatment of hip OA. While the number of studies and their methodology are still limited, the available evidence suggests safety and overall promising results, with the treatment success being inversely proportional to the severity of OA. Further high-level controlled trials are needed before drawing more definitive conclusions on the real potential of orthobiologics for the injective treatment of patients affected by hip OA.

## Figures and Tables

**Table 1:** Upper section: Kellgren–Lawrence osteoarthritis (OA) grades of the included patients; Grades I and II are classified as early OA, grade III as moderate OA, and grade IV as severe OA. Lower section: Tönnis OA grades of the included patients; Grade 0 no signs of OA, grade 1 as mild OA grade 2 as moderate OA, and grade 3 as severe OA. + study that included patients with the corresponding OA grade but did not specify the numbers; − study that did not include patients with the corresponding OA grade.

**Studies Using Kellgren–Lawrence Scale**	**Grade I**	**Grade II**	**Grade III**	**Grade IV**
Battaglia M. et al., 2011 [22]	−	4	8	8
Singh JR. et al., 2019 [24]	7	11	9	9
Battaglia M. et al., 2013 [26]	−	39	44	17
Di Sante L. et al., 2016 [28]	−	12	31	−
Doria C. et al., 2017 [29]	+	+	+	−
Villanova-López MM. et al., 2020 [30]	27	37	10	
Kraeutler MJ. et al., 2021 [31]	−	9	14	−
Palco M. et al., 2021 [32]	−	24	28	−
Emadedin M. et al., 2015 [34]	−	−	+	+
Burnham R. et al., 2021 [39]	+	+	+	+
Heidari N. et al., 2022 [41]	25	28	33	61
**Studies Using Tönnis Scale**	**Grade 0**	**Grade 1**	**Grade 2**	**Grade 3**
Sánchez M. et al., 2012 [23]	−	−	12	28
Ortiz-Declet V. et al., 2020 [25]	3	6	2	−
Mazzotta A. et al., 2022 [33]	−	11	42	43
Mardones R. et al., 2017 [35]	−	2	9	2
Rodriguez-Fontan F. et al., 2018 [36]	−	+	+	−
Dall’Oca C. et al., 2019 [37]	+	+	+	−
Whitney KE. et al., 2020 [38]	−	−	6	12
Meadows MC. et al., 2021 [40]	−	3	6	−

**Table 2:** Characteristics of PRP included studies.

Author Year	Study Design	Injective Product	Product Manufacturing and Characteristics	Injection Schedule and Amount	Patients (Sex) Age Mean + SD	Final F-up	mCMS	Results
Battaglia M. 2011 [22]	Prospective Case Series	PRP	NR	3 injections 2 weeks intervals 5 mL US guidance	20 (13 M/7 F) 52 ± 13	12 m	41	PRP injections are safe and effective in reducing pain and improving articular function and quality of life in patients affected by hip OA.
Sánchez M. 2012 [23]	Prospective Case Series	PRP	LP-PRP (PRGF) Activation: Ca chloride (10%)	3 injections 1–2 weeks intervals 8 mL US guidance	40 (27 M/12 F) 56 ± 11.9	6 m	49	PRP injections improved pain and function in a limited number of patients with severe hip OA.
Battaglia M. 2013 [26]	RCT	PRP	LR-PRP Activation: Ca chloride (10%) Plts: Increased 600% vs. WB each unit contained 6 to 8 mln plts Leukocytes: 8.3 × 10^3^/μL	3 injections 2 weeks intervals 5 mL US guidance	52 (20 M/30 F) 51 ± 12	12 m	73	IA injections of PRP are efficacious in terms of functional improvement and pain reduction but are not superior to HA in patients with symptomatic hip OA at 12-month F-up.
		HA	HMW-HA (1500 kDa) (Hyalubrix 30 mg/2 mL)	3 injections 2 weeks intervals 2 mL US guidance	52 (17M/33F) 56 ± 12			
Dallari D. 2016 [27]	RCT	PRP	LR-PRP Activation: Ca chloride (10%)	3 injections 1-week interval 5 mL US guidance	44 (20 M/24 F) NR	12 m	80	IA PRP injections offer a significant clinical improvement in patients with hip OA without relevant side effects. The addition of PRP+HA did not lead to a significant improvement in pain symptoms.
		HA	HMW-HA (1500 kDa) (Hyalubrix 30 mg/2 mL)	3 injections 1-week interval 2 mL US guidance	36 (26 M/10 F) NR			
		PRP+HA	LR-PRP + HA	3 injections 1-week interval 7 mL (5 mL PRP + 2 mL HA) US guidance	31 (12 M/19 F) NR			
Di Sante L. 2016 [28]	RCT	PRP	LP-PRP Plts: 100–150% vs. WB	3 injections 1-week interval 3 mL US guidance	21 (11 M/10 F) 71.37 ± 6.03	4 m	66	IA PRP had an immediate effect on pain that was not maintained at longer term F-up when, on the contrary, the effects of IA HA were evident.
		HA	Na-HA (30 mg/2 mL of HA with HMW 1000 to 2900 kDa)	3 injections 1-week interval 2 mL US guidance	22 (9 M/13 F) 73.62 ± 7.87			
Doria C. 2017 [29]	RCT	PRP	NR	3 injections 1-week interval 5 mL US guidance	40 (NR) 67.3 ± 5.8	12 m	68	PRP did not offer significantly better results compared with HA in patients with moderate signs of OA.
		HA	HA (Hyalubrix 15 mg/mL)	3 injections 1-week interval NR US guidance	40 (NR) 68 ± 4.6			
Singh JR. 2019 [24]	Retrospective Case Series	PRP	LP-PRP No activation	Single injection 6 mL IA + 1 mL extracapsular US or fluoroscopy guidance	36 (12 M/24 F) 66.0 ± 12.1	6 m	51	In patients with mild/moderate hip OA, PRP may provide pain relief and functional improvement for up to 6 months.
Ortiz-Declet V. 2020 [25]	Prospective Case Series	PRP	LP-PRP Plts: 2–3 times the level of WB	3 injections 1-week interval 4–7 mL US guidance	9 (4 M/5 F) 51.3 ± 9.4	12 m	61	Patients with early hip OA had significant improvements up to 12 months after PRP injections.
Villanova- López MM. 2020 [30]	RCT	PRP	LR-PRP Plts: 2.22 times the level of WB Leukocytes: 3.87 ± 2.11 × 10^3^/μL	Single injection 6 mL US guidance	38 (14 M/24 F) 61.2 ± 9.72	12 m	70	PRP is as effective and safe as those of HA for the treatment of hip OA in its initial stages.
		HA	HA (Synvisc-One^®^ 60 mg/6 mL)	Single injection 6 mL US guidance	36 (19 M/17 F) 61.1 ± 12.3;			
Kraeutler MJ. 2021 [31]	RCT	PRP	LP-PRP Activation: Ca chloride. Plts: 2–3 times the level of WB No leukocytes	3 injections 1-week interval 4–8 mL PRP No guidance	19 (8 M/10 F) 53.3 ± 8.4	24 m	79	LP-PRP resulted in an improvement in WOMAC scores and hip internal rotation at 6 months and delayed the need for THA compared with treatment with LMW-HA.
		HA	Na-HA (Supartz; 10 mg/2.5 mL)	3 injections 1-week interval 2.5 mL PRP No guidance	15 (10 M/3 F) 53.6 ± 7.6			
Palco M. 2021 [32]	Retrospective Comparative Study	LR-PRP	LR-PRP Plts: 370,000/μL Leukocytes: 4 × 10^3^/μL	2 injections 2 weeks interval 5 mL US guidance	26 (16 M/10 F) 50.62 ± 16.14	12 m	57	Both treatments are effective at reducing pain in the short to medium term. LR-PRP could be the treatment of choice due to a more marked effect over time.
		PRP +HA	Cellular Matrix A-CP-HA centrifugation	2 injections 2 weeks interval 5 mL (3 mL PRP + 2 mL HA) US guidance	26 (12 M/14 F) 64.81 ± 10.81			
Mazzotta A. 2022 [33]	Prospective Comparative Study	C-PRP	LR-PRP Activation: Ca-gluconate (10%) Plts increased by 4–5 times vs. the baseline mean plts concentration of 1000 × 10^9^/L ± 20%	3 injections 1-week interval 5 mL US guidance	50 (26 M/20 F) 47 ± 11.9	12 m	63	C-PRP is a safe approach for the treatment of patients with hip OA, with a low rate of adverse events and failures, although it provided only a mild clinical improvement comparable with A-PRP.
		A-PRP	LR-PRP Activation: Ca-gluconate (10%) Plts increased by 4–5 times vs. the baseline mean plts concentration of 1000 × 10^9^/L ± 20%	3 injections 1-week interval 5 mL US guidance	50 (34 M/16 F) 49.5 ± 12.2			

A-PRP, autologous platelet rich plasma; C-PRP, cordonal platelet rich plasma; F, female; F-up, follow-up; HA, hyaluronic acid; HMW, high molecular weight; IA, intra-articular; LMW, low molecular weight; LP-PRP, leukocyte-poor platelet rich plasma; LR-PRP, leukocyte-rich platelet rich plasma; m, months; mCMS, modified Coleman Methodology Score; M, male; NR, not reported; OA, osteoarthritis; plts, platelets; PRGF, plasma rich in growth factors; RCT, randomized controlled trials; SD, standard deviation; THA, total hip arthroplasty; US, ultrasound; WB, whole blood; WOMAC, Western Ontario and McMaster University Osteoarthritis index.

**Table 3:** Characteristics of the cell-based included studies.

Author Year	Study Design	Injective Product	Product Manufacturing and Characteristics	Injection Schedule and Amount	Patients (Sex) Age Mean + SD	Final F-up	mCMS	Results
Emadedin M. 2015 [34]	Prospective Case Series	BM-MSC	Autologous Harvest from both iliac crests Expanded Characterized for membrane markers Tested for possible microbial contamination	Single injection 10 mL Fluoroscopy guidance	6 (NR) NR	30 m	51	BM-MSC injection is safe and therapeutically beneficial in patients with hip OA.
Mardones R. 2017 [35]	Prospective Case Series	BM-MSC	Autologous Harvest form posterior iliac crest Expanded Characterized for membrane markers Tested for possible microbial contamination	3 injections 1-week interval NR No guidance	10 (5 M/5 F) 54.7	40 m	54	The IA injection of 3 consecutive weekly doses of expanded autologous BM-MSC proved to be a safe and clinically effective in patients with hip OA.
Rodriguez- Fontan F. 2018 [36]	Prospective Case Series	BMAC	Autologous Harvest from the anterior iliac crest Not expanded BioCUE Platelet Concentration System	Single injection 12 mL US or RX guidance	13 (NR); 58 ± 12.7 (also knee)	24 m	54	IA injections of BMAC were safe and demonstrated satisfactory results for the treatment of early hip OA.
Dall’Oca C. 2019 [37]	Retrospective Case Series	MF-AT	Autologous Harvest from abdominal wall adipose tissue Not expanded Lipogems^®^ system	Single injection 5–10 mL Fluoroscopy guidance Traction	6 (5 M/1 F) 52 (37–60)	6 m	42	MF-AT injection provided a significant clinical improvement in patients with early hip OA.
Whitney KE. 2020 [38]	Prospective Case Series	BMAC	Autologous Harvest from the posterior iliac crest Not expanded Hematology analysis	Single injection 6–12 mL US guidance	21 (7 M/9 F) 57.6 ± 11	6 m	57	A single BMAC injection can significantly improve subjective pain and function scores up to 6 months in patients with symptomatic hip OA.
Burnham R. 2021 [39]	Prospective Case Series	BMAC	Autologous Harvest from the posterior iliac crest Not expanded	Single injection 8–10 mL US guidance	30 (64 M/48 F) 64.1 ± 9.1	12 m	60	Hip OA treated with a single BMAC injection resulted in significant improvements in pain, disability, and quality of life with a low complication rate.
Meadows MC. 2021 [40]	Prospective Case Series	ASA	Homologous Not expanded	Single injection 4 mL US guidance	10 (5 M/4 F) 54.2 ± 6.0	12 m	53	Promising results for relief of pain and improvement in patient-reported outcomes with IA ASA in patients affected by hip OA.
Heidari N. 2022 [41]	Prospective Comparative Study	MF-AT	Autologous Harvest from abdominal wall adipose tissue Lipogems^®^ system	Single injection 6 mL US guidance	57 (21 M/36 F) 60	12 m	59	Positive role for IA injection of MF-AT + PRP as a treatment for hip OA which may be important particularly in low BMI patients where the difficulty in obtaining sufficient MF-AT.
		MF-AT + PRP	LP-PRP Activation: Ca-chloride Rich in plts	Single injection 6ml (4 mL MF-AT + 2 mL PRP) US guidance	90 (53 M/37 F) 60			

ASA, amniotic suspension allograft; BMAC, bone marrow aspirate concentrate; BMI, body mass index; BM-MSC, bone marrow mesenchymal stromal cells; F, female; F-up, follow-up; IA, intra-articular; LP-PRP, leukocyte-poor platelet rich plasma; m months; mCMS, modified Coleman Methodology Score; M, male; MF-AT, micro-fragmented adipose tissue; NR, not reported; OA, osteoarthritis; plts platelets; SD, standard deviation; US, ultrasound.

## Data Availability

Not applicable.
